# Reawakening the Intrinsic Cardiac Regenerative Potential: Molecular Strategies to Boost Dedifferentiation and Proliferation of Endogenous Cardiomyocytes

**DOI:** 10.3389/fcvm.2021.750604

**Published:** 2021-10-08

**Authors:** Chiara Bongiovanni, Francesca Sacchi, Silvia Da Pra, Elvira Pantano, Carmen Miano, Marco Bruno Morelli, Gabriele D'Uva

**Affiliations:** ^1^Department of Experimental, Diagnostic and Specialty Medicine (DIMES), University of Bologna, Bologna, Italy; ^2^Centre for Applied Biomedical Research (CRBA), University of Bologna, Bologna, Italy; ^3^National Laboratory of Molecular Biology and Stem Cell Engineering, National Institute of Biostructures and Biosystems (INBB), Bologna, Italy; ^4^Istituto di Ricovero e Cura a Carattere Scientifico (IRCCS) MultiMedica, Milan, Italy

**Keywords:** heart regeneration, direct cardiogenesis, cardiomyocyte proliferation, cardiomyocyte dedifferentiation, heart development, endogenous cardiac repair

## Abstract

Despite considerable efforts carried out to develop stem/progenitor cell-based technologies aiming at replacing and restoring the cardiac tissue following severe damages, thus far no strategies based on adult stem cell transplantation have been demonstrated to efficiently generate new cardiac muscle cells. Intriguingly, dedifferentiation, and proliferation of pre-existing cardiomyocytes and not stem cell differentiation represent the preponderant cellular mechanism by which lower vertebrates spontaneously regenerate the injured heart. Mammals can also regenerate their heart up to the early neonatal period, even in this case by activating the proliferation of endogenous cardiomyocytes. However, the mammalian cardiac regenerative potential is dramatically reduced soon after birth, when most cardiomyocytes exit from the cell cycle, undergo further maturation, and continue to grow in size. Although a slow rate of cardiomyocyte turnover has also been documented in adult mammals, both in mice and humans, this is not enough to sustain a robust regenerative process. Nevertheless, these remarkable findings opened the door to a branch of novel regenerative approaches aiming at reactivating the endogenous cardiac regenerative potential by triggering a partial dedifferentiation process and cell cycle re-entry in endogenous cardiomyocytes. Several adaptations from intrauterine to extrauterine life starting at birth and continuing in the immediate neonatal period concur to the loss of the mammalian cardiac regenerative ability. A wide range of systemic and microenvironmental factors or cell-intrinsic molecular players proved to regulate cardiomyocyte proliferation and their manipulation has been explored as a therapeutic strategy to boost cardiac function after injuries. We here review the scientific knowledge gained thus far in this novel and flourishing field of research, elucidating the key biological and molecular mechanisms whose modulation may represent a viable approach for regenerating the human damaged myocardium.

## Toward the Direct Stimulation of Cardiomyocyte Proliferation for Heart Regeneration

Heart failure, consisting in the inability of the heart to pump enough blood to meet the body's needs, is a prominent cause of death worldwide and often occurs as a result of severe cardiac injuries, such as those induced by myocardial infarction [reviewed by Savarese and colleagues (1)]. Besides left ventricular assist devices and heart transplant, which is the most curative approach, yet with severe limitations (scarcity of donors, extremely high costs, immune response, and organ rejection, etc.), currently available therapies are mainly based on pharmacological treatments for slowing down disease progression and reducing symptoms. However, none of these treatments can reverse the progression of the disease or cope with the underlying conspicuous loss of cardiac muscle cells (cardiomyocytes) that are replaced by fibrotic scar tissue. During the last decades, scientific studies based on transplantation of adult stem cells, isolated from skeletal muscle, bone marrow, blood, or fat tissue, have been carried out with the hope to replenish lost or damaged cardiomyocytes, restoring cardiac function. Unfortunately, these approaches demonstrated modest beneficial effects on heart function most probably attributable to paracrine factors rather than the generation of new cardiac muscle cells [reviewed by Tzahor and Poss (2) and Sadek and Olson (3)]. Moreover, although a population of lineage negative c-kit+ cardiac stem cells was initially reported to give rise to all major cardiac cell types, including cardiomyocytes (4), more recent lineage tracing studies based on tamoxifen-inducible Cre-LoxP technology unveiled that newly cardiomyocytes generated from c-kit+ cells are extremely rare, irrelevant in terms of cardiomyocyte regeneration, despite abundantly contributing to the generation of endothelial cells (5) [reviewed by Passier and colleagues (6) and Chien and colleagues (7)].

During the last two decades, the attention of many research groups has shifted toward the possibility to regenerate the damaged heart by reawakening the intrinsic regenerative potential. Indeed, studies of the animal kingdom have enlightened the amazing ability of some animals to regenerate themselves. Hydra, planarians, and lower vertebrates, such as salamanders, frogs, and fishes, can trigger complex repair mechanisms, totally or partially restoring missing or damaged tissues and organs, such as limbs, retinas, eye lenses, spinal cords, tails, and even the heart. These astonishing observations have led to intense scientific investigations in cardiac regenerative medicine, aiming at developing innovative therapeutic strategies suitable for humans. Specifically, studies in the zebrafish model at the adult stage unveiled its ability to efficiently regenerate the damaged cardiac tissue, achieving complete scar resolution and regeneration of lost cardiomyocytes within 2 months after surgical resection of 20% of the ventricular myocardium (8). This striking self-healing property emerges even after more severe cardiac damages, such as cardiomyocyte-specific depletion of 60% of the ventricular myocardium (9), and cryoinjury-induced lesions (10, 11). Interestingly, genetic labeling of differentiated cardiomyocytes with fluorescent markers highlighted that cardiac muscle cells generated post-injury derive from the proliferation of endogenous cardiomyocytes. In this process, transient and partial dedifferentiation of cardiomyocytes has been documented, as manifested by cardiomyocyte detachment from one another, sarcomere disassembly, loss of Z-line structure, and expression of fetal genes (12, 13). Unlike zebrafish, for a long-time, the mammalian heart has been considered non-regenerative because of its injury-induced replacement of dead muscle cells with fibrotic tissue and its inability to restore the reduced contractile function after major injuries. Despite adult mammals fail in regenerating their heart, cardiac regeneration appears to be quite robust during prenatal and early postnatal stages. Indeed, mammalian fetuses can compensate for a loss of about half of cardiomyocytes (14, 15). Newborn mice can robustly regenerate their heart following resection of 15% of the ventricular apex within 2 months, by inducing the proliferation of pre-existing cardiomyocytes, as assessed by lineage tracing analyses and staining of cell cycle markers (16, 17). A complete cardiac regeneration process has also been documented in newborn mice following induction of myocardial infarction by ligation of the left anterior descending artery (18). The cardiac regenerative ability at the neonatal stage has also been documented in large mammals. For example, myocardial infarction in 1 or 2-days-old swine, is followed by cardiac tissue replacement achieved by dedifferentiation and proliferation of pre-existing cardiomyocytes in the border zone (19, 20). It has also been reported the astonishing clinical case of a newborn child undergoing a rapid functional cardiac recovery after myocardial infarction, although it was not possible to assess if the observed recovery was due to bona fide regeneration or reversible functional impairment (21). Importantly, cardiomyocyte regenerative potential in the mouse model dramatically decreases during the first week of postnatal life; consequently, severe cardiac injuries evolve in permanent scarring and impair heart function (16). This decline was suggested to start already 2 days after birth (22). Similar observations were documented in larger mammals during the early postnatal period. For example, swine begin losing cardiomyocyte regenerative ability at postnatal day 3 and more pronouncedly at later developmental stages (postnatal day 7 and 14), undergoing extensive cardiac fibrosis and not recovering cardiac function after injury (19, 20).

In this review we first describe how mammalian cardiomyocyte cell cycle activity is regulated during prenatal and postnatal life, with particular emphasis on the early postnatal period, when most cardiomyocytes become bi/multi-nucleated or polyploid, withdraw from the cell cycle, and continue to grow in size (hypertrophic growth), consequently losing the regenerative potential. Then, we review the major changes occurring at birth and in the immediate postnatal period, along with systemic, micro-environmental, intracellular stimuli influencing the proliferative ability of endogenous cardiomyocytes, whose manipulation is a promise for enhancing cardiomyocyte regeneration and boosting cardiac function in heart failure patients.

## Developmental Regulation of Cardiomyocyte Cell Cycle Activity

In zebrafish, cardiomyocytes are predominantly mononucleated and diploid throughout life and retain pronounced proliferative capacity (23, 24).

**Figure 1:**
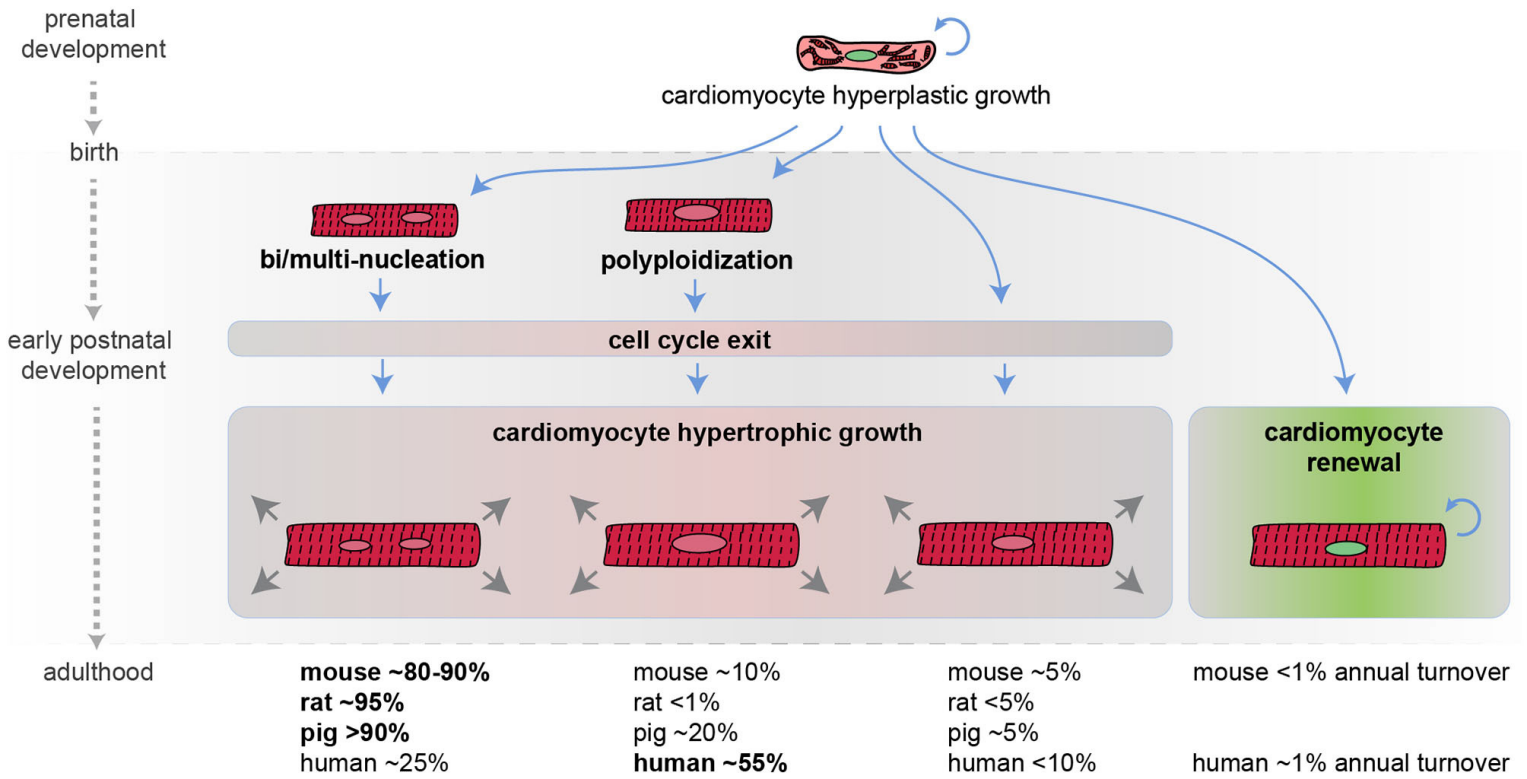
Developmental regulation of cardiomyocyte cell cycle activity in mammals. Schematic representation of mammalian cardiomyocyte growth in prenatal and postnatal life. Most cardiomyocytes during the early postnatal period become bi/multi-nucleated and/or polyploid, withdraw from the cell cycle, and continue to grow in size (hypertrophic growth). An approximate percentage of bi/multi-nucleated, polyploidy, and diploid cardiomyocytes in different mammalian species at the adult stage is provided (it may not add up to 100% because cardiomyocytes can have several polyploid nuclei and because these values are derived from different reports) along with the estimated cardiomyocyte annual turnover.

In contrast, cardiomyocyte cell cycle activity and nucleation in mammals are strictly connected to the developmental stage (Figure 1). During embryonic and fetal development heart growth in mammals is characterized by the increase in the number of cardiomyocytes. Importantly, genetic fate mapping in the mouse model, allowing the identification of the temporal sequence during which the lineage segregation between cardiomyocytes and non-myocytes takes place, unveiled that non-myocytes, which include stem cell populations, contribute to new cardiomyocyte generation exclusively in the early embryonic development (25). Starting from mid-gestation, pre-existing cardiomyocytes become the predominant source of cardiomyocyte replacement in physiological mammalian cardiac development (25). As further detailed later in this review, multiple signaling pathways were shown to play a key role in cardiomyocyte proliferation during prenatal life and, in some cases, their manipulation can partially reactivate the cardiac regenerative ability in the adult stage.

In the mouse model, during the first week after birth, the majority of cardiomyocytes undergo DNA synthesis and karyokinesis (nuclear division), without proceeding to cytokinesis (cytoplasm division), thus resulting in binucleation (two diploid nuclei per single cell) (26, 27) [reviewed by Soonpaa and Field (28)]. Specifically, on postnatal day 2 most mouse cardiomyocytes are mononucleated. On postnatal day 3 mouse binucleated cardiomyocytes raise to ~17% and reach the adult level of ~80–90% by day 11 (26) [reviewed by Derks and Bergmann (29)]. Similarly, in rats, the percentage of binucleated cardiomyocytes, which is around 3–4% in the first 3 days of postnatal life, increases at ~17% on postnatal day 4 and reaches the adult level of ~90% by day 12 (30) [reviewed by Derks and Bergmann (29)].

In humans, during the early postnatal period, the majority of cardiomyocytes undergo DNA synthesis without karyokinesis, resulting in polyploidization (single tetraploid nuclei) (31). Other large mammals, such as swine, undergo primarily multinucleation and to a less extent polyploidization (32) [reviewed by Derks and Bergmann (29)].

The time at which cardiomyocytes become bi/multi-nucleated or polyploid is coincident with the time when mammals lose their regenerative potential [reviewed by Derks and Bergmann (29) and Gan and colleagues (33)]. In support of a causal relationship between multinucleation/polyploidy and loss of cardiac regenerative ability, enforced cardiomyocyte polyploidization has been demonstrated to reduce cardiomyocyte proliferation and to represent a barrier to heart regeneration in the zebrafish model (24). Importantly, during the early postnatal development, the vast majority of mammalian cardiomyocytes also exit from the cell cycle. As a consequence, the number of postnatal cardiomyocytes does not increase in mammals during postnatal life (34), and further growth of the heart is achieved by increasing cardiomyocyte size, a phenomenon known as hypertrophic growth.

Historically, adult human cardiomyocytes were considered completely unable to divide. However, this belief has been disproved in 2009. Indeed, the analysis of the integration of ^14^C generated by nuclear bomb tests during the Cold War allowed to precisely estimate cardiomyocyte renewal in adult humans. This study detected 1% annual cardiomyocyte turnover at the age of 25, declining to 0.3% at the age of 75. Based on these data, it is therefore estimated that fewer than 50% of cardiomyocytes are physiologically exchanged during the course of life (34). Even though the adult cardiomyocyte renewal rate is extremely low, definitely insufficient to pursue a successful regenerative process after major injuries, this remarkable observation suggests that increasing the rate of adult cardiomyocyte proliferation may represent a novel strategy for cardiac regeneration.

## Molecular Strategies for Cardiomyocyte Regeneration

**Figure 2:**
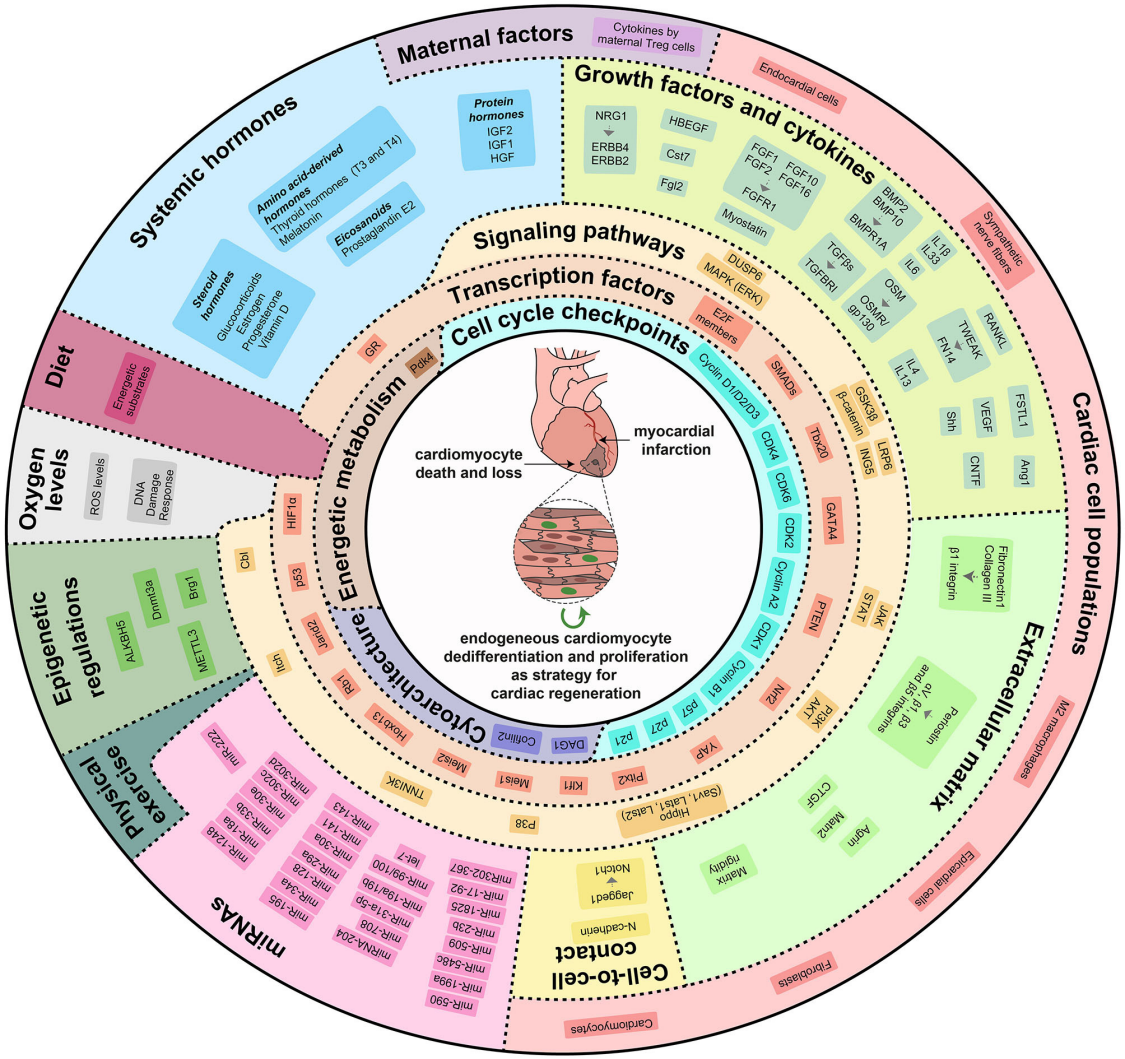
Cardiac regenerative strategies based on direct stimulation of cardiomyocyte dedifferentiation and proliferation. Modulation of external, systemic, micro-environmental, and intrinsic molecular mechanisms can re-activate cardiomyocyte proliferative and regenerative potential. Locally produced growth factors and cytokines, extracellular matrix rigidity and components, direct cell-to-cell contacts, maternal factors, systemic hormones, oxygen levels, physical exercise, miRNAs and epigenetic regulations modulate a variety of signaling pathways and transcription factors that control cardiomyocyte dedifferentiation and proliferation by regulating cell cycle checkpoints, cytoarchitectural organization and energetic metabolism.

Immediately after birth, a complex reorganization of the cardiovascular system occurs. Recent studies have unveiled that the adaptation from intrauterine to extrauterine life driven by the sudden lack of exposure to circulating maternal factors, the increase in oxygen levels, the increase in heart workload, as well as changes of systemic, microenvironmental, and intracellular stimuli, lead to maturation of cardiomyocyte cytoarchitecture, switch in energetic metabolism from glycolysis to fatty acid oxidation and cell cycle withdrawal during the early postnatal period, concurring to postnatal loss of cardiac regenerative ability. Importantly, the manipulation of specific molecular mechanisms has been demonstrated to be sufficient for inducing cardiomyocyte proliferation and heart regeneration upon injury (Figure 2).

### Cell Cycle Checkpoints

**Figure 3:**
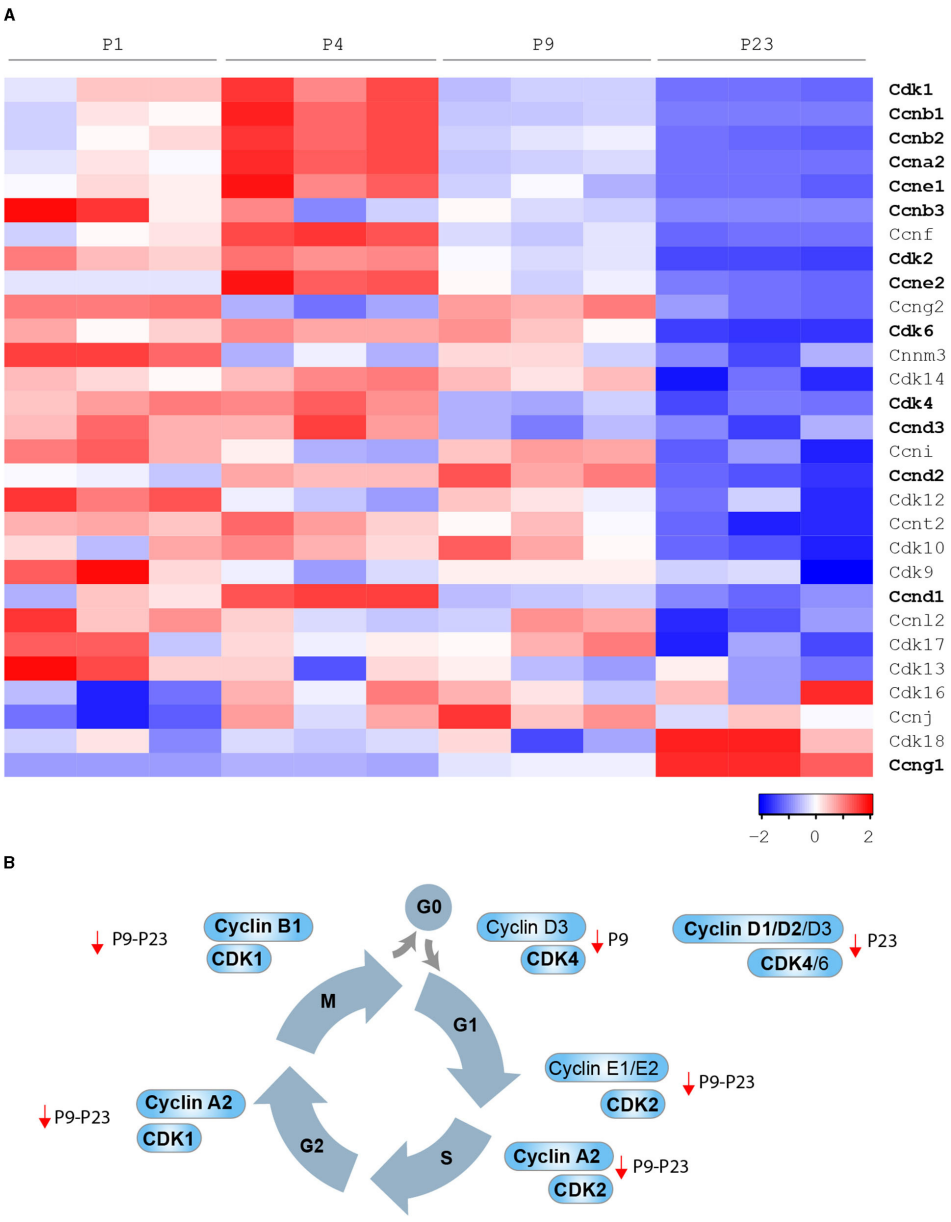
Developmental regulation of cardiomyocyte cell cycle in mammals. **(A)** Cyclins and Cdks expression levels by bioinformatic analysis of the gene expression profile of the mouse heart at different developmental stages [P1, P4, P9, and P23 from Talman et al. (39)]; **(B)** Cyclins and CDKs whose modulation has been demonstrated to be sufficient to induce postnatal cardiomyocyte cell cycle progression (in bold cell cycle factors that were reported to induce adult cardiomyocyte regeneration after major injuries).

Multiple regulators of cell cycle checkpoints, including cyclins, cyclin-dependent protein kinases (CDKs), CDK-activating kinases (CAKs), and CDK inhibitors (CKIs) were documented to regulate cardiomyocyte cell cycle activity during prenatal and postnatal development. Cyclin/CDK function is mainly regulated by post-transcriptional or post-translational modifications. However, cardiac mRNA and protein levels of several cyclins/CDKs were documented to decrease during postnatal development (35–38) (bioinformatic analysis in Figure 3A) and, in several cases, their overexpression was sufficient to induce postnatal cardiomyocyte cell cycle activity (Figure 3B). Thus, the decline in expression levels of specific cyclins/CDKs contributes to mammalian cardiomyocyte cell cycle blockage in postnatal life.

D-type cyclins, when complexed with CDK4 or CDK6, drive cell cycle re-entry (transition from G0 to G1 phase). High protein levels of D-type cyclins, CDK4 and CDK6, have been reported in the fetal heart, dramatically declining in the early postnatal and adult stage (26). In agreement, we observed that cardiac mRNA levels of cyclin D3 (Ccnd3) and CDK4 significantly decline in the early postnatal period (postnatal day 9–P9), whereas cardiac mRNA levels of cyclin D1 (Ccnd1), cyclin D2 (Ccnd2), and CDK6 decline in the subsequent postnatal developmental step (postnatal day 23–P23) (see Figures 3A,B). **Cyclin D1** overexpression has been reported to induce abnormal multinucleation (35). The impairment of its nuclear import in differentiated cardiomyocytes, in part due to the accumulation of CDK inhibitor p27, has emerged as a barrier that prevents postnatal cardiomyocyte proliferation (40). Indeed, overexpression of Skp2 ubiquitin ligase, which triggers the degradation of p27, enhances the mitogenic effect mediated by nuclear-targeted cyclin D1 (D1NL)/**CDK4** and improves cardiac function after myocardial infarction (40). Cardiac-specific overexpression of cyclin D1, **cyclin D2** or **cyclin D3** results in increased DNA synthesis of mammalian cardiomyocytes in adult mice (38). However, myocardial damage reduces the pro-proliferative effect of transgene-encoded cyclin D1 and D3 by inducing their cytoplasmatic accumulation. Importantly, the cardiac injury does not induce cytoplasmatic accumulation of transgene-encoded cyclin D2, which indeed has been documented to maintain persistent cell cycle activity in cardiomyocytes and to trigger infarct regression (38).

E-type cyclins, in association with CDK2, control the G1 phase of the cell cycle and are known to initiate the assembly of the pre-replication complex. Cardiac protein levels of CDK2 are drastically reduced from fetal to adult stage (38). In this regard, we observed that cardiac mRNA levels of CDK2 significantly decline in the early postnatal period (P9) (see Figures 3A,B). Chemical inhibition of **CDK2** suppresses DNA synthesis of neonatal cardiomyocytes (41), whereas its overexpression increases the number of smaller mononuclear cardiomyocytes in adult mice (42). We also noticed that cardiac mRNA levels of cyclin E1 (Ccne1) and cyclin E2 (Ccne2) significantly decline in the early postnatal period (P9) and the subsequent developmental step (P23) (see Figures 3A,B). However, the role of E cyclins in cardiomyocyte proliferative and regenerative ability is currently unexplored.

A-type cyclins are required for entry into S phase (in association with CDK2) or into M phase (in association with CDK1). Interestingly, cardiac protein levels of cyclin A1 and A2 were shown to decline in postnatal development, and very low levels of CDK1 have been reported in the adult heart (37, 43). Consistently, we observed that mRNA levels of cyclin A2 (Ccna2) and CDK1 significantly decline during the early postnatal period (P9, see Figures 3A,B), whereas cyclin A1 (Ccna1) mRNA was generally poorly expressed in postnatal life (P1-P4-P9 and P23, **data not shown**). Adenoviral overexpression of **cyclin A2** has been documented to enhance the endogenous regenerative mechanism after myocardial infarction by the generation of new cardiomyocytes in the infarct and border zones, along with improved cardiac function and reduced collagen/muscle density ratio (37, 44, 45). The injection of adenovirus encoding cyclin A2 into the peri-infarct myocardium has been reported to induce cardiomyocyte mitosis, to decrease fibrosis and to boost cardiac function in larger preclinical models (swine) (46).

B-type cyclins in association with CDK1 positively regulate the transition from G2 to M phase. Interestingly, cardiac levels of cyclin B1 and CDK1 protein were documented to be dramatically reduced from fetal to adult stage (36). In line, we observed that cyclin B1 (Ccnb1) mRNA levels significantly decline in the early postnatal period (P9), whereas cardiac mRNA levels of cyclin B2 (Ccnb2) and cyclin B3 (Ccnb3) decline later on during postnatal development (P23) (see Figures 3A,B). Forced expression of **cyclin B1** and CDC2 (human homolog of **CDK1**) increases the number of neonatal and adult rat cardiomyocytes *in vitro* (36).

Interestingly, overexpression of a combination of **CDK4-cyclin D1** and **CDK1-cyclin B1** complexes in adult cardiomyocytes has been documented to promote a high rate (~15%) of cardiomyocyte proliferation and to contribute to heart regeneration after coronary artery ligation in adult mice (47). The replacement of CDK1-cyclin B1 overexpression through a pharmacological approach, based on the administration of inhibitors of Wee1 (CDK1-inhibitor) and transforming growth factor-β (TGF-β), proved to be an alternative way to unlock adult cardiomyocyte replicative ability (47).

Oppositely, cardiac mRNA and protein levels of cyclin G1 (Ccng1) increase during the early postnanal development and in the adult stage (48) (see Figure 3A). However, unlike the other cyclins described above, cyclin G1 has been linked to the onset of postnatal cardiomyocyte polyploidization and multinucleation. Indeed, its overexpression in primary neonatal rat cardiomyocytes promotes entry in S-phase (uptake of ^3^H-thymidine), however reducing the number of cytokinetic events (Aurora B immunostaining), and thus resulting in an increase of polynucleated cells. In contrast, the knockout of cyclin G1 prevents the increase of cardiomyocyte multinucleation in response to pressure overload and hypertrophy (48).

Cyclin-dependent Kinase Inhibitors (CKIs**)**, including **p21**, **p27**, and **p57**, were suggested to contribute to cardiomyocyte cell cycle withdrawal as the heart ages. Manipulation of their physiological expression by siRNA delivery has been documented to stimulate cytokinesis of neonatal cardiomyocytes and progression to the S phase of post-mitotic cells without DNA damage or apoptosis (49).

### Maternal Factors

In mammals, during fetal life, the placenta supplies all physiological needs. A major change occurring after birth, besides major hemodynamic and biochemical events, is the sudden lack of exposure to the maternal circulation. Intriguingly, exposure to the serum of pregnant animals has been reported to promote neonatal cardiomyocyte proliferation (50), suggesting that mother's serum factors might be involved. Further analyses unveiled a role for **regulatory T cells** (**Tregs**) in this process (50). Tregs are physiologically expanded during pregnancy and they are crucial for the suppression of allogenic responses toward the fetus (51). Endogenous Tregs have been found able to support cardiomyocyte hyperplasia and the increase in heart size physiologically occurring during pregnancy (50). Furthermore, Treg injection at the site of myocardial infarction has been documented to promote cardiomyocyte replication and heart regeneration (50). The effect appears mediated by a group of six cytokines secreted by Tregs, namely **TNF superfamily member 11 (Tnfsf11 or RANKL)**, **Interleukin-33 (IL33)**, **Insulin-like growth factor 2 (IGF2)**, **Cystatin F (Cst7)**, **Fibrinogen-like 2 (Fgl2)**, and **Matrilin2 (Matn2**) (50). Indeed, the production of the six factors by adenoviral vectors is sufficient to induce neonatal cardiomyocyte proliferation *in vitro*, as well as cardiomyocyte proliferation and heart regeneration *in vivo* in adult mice (50).

### Oxygen Levels

During fetal stages, the oxygenated maternal blood mixes with poorly oxygenated blood within the placental space. Thus, the oxygen content supplied to the fetus is lower than the maternal uterine arterial blood, resulting in the fetus living in a more hypoxemic environment. One of the major adaptations that mammals must face during the transition from fetus to newborn, when pulmonary circulation starts, is the exposure to a more oxygenated environment. Importantly, the change in oxygen concentration has been demonstrated to impact on cardiomyocyte proliferative and regenerative ability. In contrast to anoxia, which is reported to impair cardiomyocyte proliferation (52), the exposure to mild hypoxic conditions (15% O2) in neonatal mice is sufficient to enhance cardiomyocyte mitogenesis, protecting cells from oxidative stress (53). In line, hypoxemia exposure to 2-months-old mice, by a gradual reduction in inspired oxygen until 7%, is sufficient to facilitate the proliferation of pre-existing cardiomyocytes and heart regeneration after myocardial infarction, thus improving left ventricular systolic function (54). Importantly, intermittent hypoxia-hyperoxia appears to facilitate the rehabilitation of patients with coronary artery disease (55). In contrast, hyper-oxidative (100% O2) exposure is responsible for oxidative DNA damage and decreased cytokinesis in mouse models (53).

During the first week of postnatal life, the increase in oxygen levels contributes to the decline in heart regenerative ability by triggering oxidative energetic mitochondrial metabolism (further described in the “*Energetic metabolism*” section) and by inducing **reactive oxygen species (ROS), oxidative DNA damage**, and **DNA damage response (DDR)** (53). Indeed, ROS scavenging, or inhibition of DDR is sufficient to extend the postnatal proliferative window of cardiomyocytes, whereas ROS production shortens it (53).

Clinical studies on cyanotic congenital heart disease infants suggest that the hypoxic condition reflects an increased mitotic potential of cardiomyocytes (56). Ablation of **Hypoxia-inducible factor 1-alpha (HIF1α)**, a major mediator of the hypoxic response, reduces fetal cardiomyocyte proliferation and results in ventricular hypoplasia (57). Moreover, by lineage-tracing studies employing a tamoxifen-inducible Cre fused to the oxygen-dependent degradation domain of HIF1α, it has been unveiled that a population of hypoxic cycling cardiomyocytes contributes to the slow cardiomyocyte turnover occurring in the adult mammalian heart (58). Interestingly, a downstream target of HIF1α, named Zinc finger E-box-binding homeobox 2 (ZEB2), has been recently demonstrated to be enriched in injured cardiomyocytes of zebrafish models. Its overexpression improves cardiomyocyte survival and cardiac function, as well as angiogenesis following cardiac damage (59), however, the role of ZEB2 on cardiomyocyte proliferation has not been explored.

### Energetic Metabolism

In zebrafish, proliferating cardiomyocytes in the border zone of the wounded heart, where cardiomyocyte dedifferentiation mainly occurs, switch their metabolism from oxidative phosphorylation to glycolysis, as manifested by reduced mitochondrial genes and increased glycolytic genes (60). This process was reported to be induced by Neuregulin-1/Erbb2 signaling (60). Importantly, inhibition of glycolysis after cardiac injury impairs cardiomyocyte proliferation in adult zebrafish (60).

In mammals, during the prenatal period, glucose is the main source of energy for cardiomyocytes, and anaerobic glycolysis is the primary energetic route. With the transition to extrauterine life, the low-fat and high glucose supply in the umbilical blood is replaced by the high fat, low glucose diet of the mother's milk. As a consequence of the increase in oxygen levels (due to the opening of the pulmonary circulation) and the shift in substrate utilization (from glucose to fatty acids), cardiomyocytes experience a profound change in the energetic metabolism during the early postnatal development, with a rewiring from anaerobic cytoplasmic glycolysis to mitochondrial-dependent oxidative phosphorylation [reviewed by Piquereau and Ventura-Clapier (61)]. This transition is driven by the upregulation of **genes involved in fatty acid metabolism and oxidative phosphorylation**, and the downregulation of **glycolytic genes** (62). The maturation of cardiomyocyte cytoarchitecture occurring in the early postnatal development (further described in the “*Cytoarchitectural organization*” section), is coupled with a transition from sparse to dense and well-organized mitochondrial clusters and a more efficient energy transfer system from mitochondria to sarcomere structures [reviewed by Piquereau and Ventura-Clapier (61)]. Although mitochondrial oxidative metabolism is a more efficient energy production to face the increasing cardiomyocyte needs of the postnatal heart, recent insights have demonstrated that the glycolysis-to-fatty-acid-oxidation metabolic switch concurs to the postnatal loss of cardiomyocyte proliferative and regenerative ability. In this regard, mitochondrial maturation has been suggested as a mediator of cardiomyocyte cell cycle arrest (53). Further, fetal cardiomyocytes were found more mitotic and with delayed maturation when exposed to maternal hyperglycemia (63). Administration of a fat deficient diet is sufficient to increase the generation of new cardiomyocytes in young mice, even though no differences were then observed after 10 weeks of age (64). In addition, cardiac-specific ablation, or pharmacological inhibition of **pyruvate dehydrogenase kinase 4 (PDK4)**, which physiologically inhibits mitochondrial pyruvate dehydrogenase thus improving cardiac fatty acid oxidation, induces cardiomyocyte proliferation and improves cardiac function after myocardial infarction (64).

### Cytoarchitectural Organization

During the early postnatal heart development in mammals, cardiomyocytes experience a profound maturation of the **cytoarchitecture organization** that, along with an increase in matrix rigidity (described in “*Extracellular matrix*” section), is essential to adequately respond to the increased workload of the extrauterine life [reviewed by Guo and Pu (65)]. Specifically, the loss of cardiac regenerative potential is coupled with an increase in cardiomyocyte cell size, and a shift of the cardiomyocyte cytoarchitectural structure from loose spatial organization to highly organized and efficient sarcomere units, characterized by the alignment of Z-lines, distinguishable M-lines and switch from fetal to adult sarcomere isoforms [reviewed by Guo and Pu (65)]. Importantly, the sarcomere apparatus occupies a large proportion of the cell, and the rigid sarcomere structure of adult cardiomyocytes makes them more refractory to cytokinesis. Interestingly, spontaneous heart regeneration occurring in injured zebrafish and neonatal mice appears coupled with sarcomere disassembly (12, 13, 16). Some regulators of the remodeling of the cardiomyocyte architecture have been demonstrated to affect cardiomyocyte proliferative and regenerative ability [reviewed by Ali and colleagues (66)], including actin-depolymerizing factor **Cofilin 2** (67) (further described in the “*miRNA*” section) and dystroglycan **DAG1** (68) (further described in the “*Extracellular matrix*” section), which anchors the cardiomyocyte cytoskeleton to the extracellular matrix. Furthermore, unlike zebrafish and newts, which preserve intact centrosomes throughout life, **centrosome integrity** is lost shortly after birth in mammals and has been described to contribute to postnatal cardiomyocyte G0-G1 cell cycle arrest (69).

### Cardiac Cell Populations

After cardiac injuries in lower vertebrates or neonatal mammals, a series of cellular events take place to trigger the regeneration of the damaged tissue. An inflammatory phase driven by recruited leukocytes starts immediately after the injury. In this regard, endogenous **macrophages** have emerged as essential players for heart regeneration in lower vertebrates and neonatal mice. Indeed, macrophage depletion impairs myocardium regeneration following injuries, leading to scar formation in zebrafish (70) and neonatal mice (71, 72). Secretion of Oncostatin M (OSM, described in the “*Growth factors and cytokines*” section) by macrophages/monocytes appears to be essential for cardiomyocyte proliferation during neonatal heart regeneration (73). In addition, the positive effect exerted by hypoxia exposure on cardiomyocyte proliferation (discussed in the “*Oxygen levels*” section), has been suggested to be dependent on an increase in the number of resident macrophages (56). In contrast to the neonatal stage, adult mammalian hearts mainly undergo repair processes based on scarring and fibrosis, mostly as a result of the interaction between infiltrating immune cells (including macrophages) and fibroblasts [reviewed by Chen and colleagues (74)]. The paradoxical role of macrophages, triggering cardiac regeneration in lower vertebrates and neonatal mammals, and maladaptive remodeling in injured adult mammals, has been a matter of investigation. In this regard, neonatal mice in response to cardiac injuries have been shown to expand a population of embryonic-derived resident cardiac macrophages with a pro-reparative (**M2**) polarization phenotype, which generates minimal inflammation and secretes numerous soluble factors that facilitate cardiomyocyte proliferation (71, 72). In contrast, adult mice in response to cardiac injuries expand monocyte-derived macrophages with an inflammatory (M1) phenotype, which lack regenerative properties (71, 72). Inline, M2 compared to M1 macrophage-conditioned media has been shown to upregulate neonatal cardiomyocyte proliferation and to suppress myofibroblast-induced differentiation via secretion of the anti-inflammatory cytokine **IL4 (Interleukin 4)** *in vitro* (75). However, the potential cardiac regenerative role of IL4 administration has not been further explored thus far. Moreover, administration of the anti-inflammatory cytokine IL10 (76) or BMP7 (bone morphogenetic protein 7) [reviewed by Aluganti Narasimhulu and Singla (77)] has been reported to improve cardiac remodeling after myocardial infarction by stimulating M2 macrophage polarization, although their potential impact on cardiomyocyte proliferation has not been analyzed. Thus, manipulation of macrophage lineages and/or polarization, or their secreted factors, may represent a viable strategy for cardiac regeneration.

The initial injury-induced inflammatory response in zebrafish and neonatal mice is accompanied by the activation of the endocardium and epicardium, which together with cardiac fibroblasts, repair the tissue and support its regeneration by inducing cardiomyocyte proliferation. **Endothelial cells** migrate into the apical thrombus early after cardiac damage, develop into functional arteries, and precede cardiomyocyte ingrowth during mammalian heart regeneration (78). The pro-proliferative and pro-regenerative effect of endothelial cells is likely due to paracrine factors, such as NRG1 (further described in the “*Growth factors and cytokines*” section). Activated **epicardial cells** also secrete signals with the potential to influence cardiomyocyte proliferation and heart regeneration, including BMPs, TGFbs, SHH and IGFs [reviewed by Cao and Poss (79)] (further described in the “*Growth factors and cytokines*” section). In contrast to adult cardiac fibroblasts that are known to promote myocyte hypertrophy, embryonic **cardiac fibroblasts** have been reported to enhance cardiomyocyte replication in co-culture experiments (80). This effect appears to reside in fibroblast-secreted factors, such as the extracellular matrix components fibronectin1 and collagen III as well as the growth factor HBEGF (80) (further info available in the “*Extracellular matrix*” and “*Growth factors*” sections).

Innervation also plays a key role in cardiac regeneration. In this regard, **sympathetic nerve fibers** re-grow and fully reinnervate during the spontaneous cardiac regeneration in neonatal mice (81). Importantly, denervation, achieved by pharmacological ablation of cholinergic signaling, restrains heart regeneration by inhibiting cardiomyocyte cell cycle activity in zebrafish and neonatal mice (81, 82). Interestingly, neonatal sympathetic lesions result in increased expression of Meis1 (83), a transcription factor involved in postnatal cardiomyocyte cell cycle arrest (further described in the “*Transcription factors*” section). Administration of Neuregulin-1 (NRG1) and Nerve Growth Factor (NGF) have been shown to partially rescue denervated hearts, enhancing cardiac regeneration post-injury. However, unlike NRG1, NGF is not able to directly promote the proliferation of cultured cardiomyocytes (82).

### Growth Factors and Cytokines

A wide spectrum of mitogens sustains cardiomyocyte proliferation during prenatal development. Administration of these factors has been investigated as a strategy to restore cardiomyocyte mitogenic potential, reminiscent of the embryonic stage. Nowadays, different growth factor ligands and receptors have been found able to induce adult cardiomyocyte cell cycle re-entry and proliferation and to achieve substantial improvements in terms of cardiac tissue regeneration.

**Fibroblast growth factors (FGFs)** act as paracrine or endocrine signals in heart development, health, and disease, exerting biological activities by binding to cell surface FGF receptors (FGFRs) [reviewed by Itoh and colleagues (84) and Khosravi and colleagues (85)]. Several FGF members, including FGF9, FGF10, FGF16, and FGF20 were shown to induce cardiomyocyte proliferation during embryonic/fetal development (86–88). Importantly, the ability of some FGFs in inducing postnatal cardiomyocyte replication and cardiac regeneration has also been documented. Administration of **FGF1**, alone and more pronouncedly in combination with an inhibitor of mitogen-activated protein kinase (p38), has also been demonstrated to induce neonatal and adult rat cardiomyocyte proliferation *in vitro* (89) as well as *in vivo* after myocardial infarction in adult rats, resulting in reduced scar formation and improved cardiac function (90). **FGF10** has been reported to trigger cell cycle re-entry of adult cardiomyocytes (86); however, its delivery as a strategy for adult cardiac regeneration has not been evaluated thus far. The role of **FGF16** in the regulation of postnatal cardiomyocyte replication is currently debated. Cardiac levels of FGF16 have been shown to increase in early postnatal life (91). However, in contrast to the documented positive role on cardiomyocyte proliferation during heart development (87, 88), FGF16 administration to neonatal cardiomyocytes does not influence their proliferation and even abrogate FGF2-induced cell cycle re-entry (91). Nevertheless, cardiac-specific FGF16 overexpression has been shown to improve cardiac function and cardiomyocyte replication after cryoinjury in a GATA4-knockout mouse model (92). Intriguingly, a decrease in expression levels and an isoform switching of type 1 fibroblast growth factor receptor **FGFR-1** have been reported in early postnatal life. Consistently, FGFR-1 overexpression has been shown to enhance the proliferation of postnatal rat cardiac myocytes, which appears to be dependent on **FGF2**, since its neutralization with antibodies inhibits the proliferative response (93).

**Neuregulin-1 (NRG1)** is a growth factor, mainly produced by endothelial cells and acting in cardiomyocytes via its tyrosine kinase receptors **ERBB4** and **ERBB2**. NRG1/ERBB4/ERBB2 signaling axis is essential for heart development (94–96). In zebrafish, NRG1 is sharply induced in perivascular cells after cardiac damage and inhibition of its co-receptor ERBB2 disrupts cardiomyocyte proliferation in response to injury (97). In mice, administration of NRG1 has been shown to induce adult cardiomyocyte proliferation and heart regeneration (98). Administration of NRG1 moderately improved cardiac function in heart failure patients in phase I and phase II trials (99–101). However, it has been observed that its mitogenic effect in mammals is more pronounced during the neonatal period than in later postnatal development and in adulthood (102, 103), due to the decline in cardiac levels of ERBB2, which is necessary to transduce the mitogenic signaling of NRG1 (102). Thus, combinatorial strategies of NRG1 with ERBB2 overexpression or ERBB2 inducing factors should be further explored. Indeed, transient induction of ERBB2 signaling in cardiac muscle cells of juvenile and adult mice is sufficient to robustly induce cardiomyocyte dedifferentiation and proliferation and to trigger heart regeneration following myocardial infarction (102). Analysis of ERBB2 downstream players mediating these effects in cardiomyocytes revealed the involvement of ERK, AKT, and GSK3β/β-catenin pathways (102). More recently, ERBB2 signaling has been shown to lead to phosphorylation of YAP in ERK-dependent and Hippo-independent manner (104). Interestingly, **HBEGF**, a growth factor that activates ERBB4 and the cognate EGF receptor (EGFR), has been shown to induce mammalian cardiomyocyte proliferation (80) (described in the “*Cardiac cell populations*” section).

**Bone morphogenetic proteins (BMPs)** are multi-functional growth factors belonging to the transforming growth factor beta (TGFβ) superfamily. BMPs play a key role in multiple steps of cardiac development, including differentiation of cardiomyocytes from mesoderm, cardiomyocyte growth and ventricular trabeculation [reviewed by Vanwijk and colleagues (105)]. Spatially resolved RNA sequencing of regenerating zebrafish heart unveiled that BMP signaling is activated in the border zone of the damaged myocardium, as manifested by expression of BMP ligands (BMP2 and BMP7), receptors (Bmpr1aa), and activation of downstream **SMAD** players (Smad 1, 5, and 8) (106). Importantly, BMP signaling is essential for injury-induced cardiomyocyte proliferation in zebrafish (106). In particular, a loss-of-function mutation in Bmpr1aa reduces cardiomyocyte proliferation and heart regeneration (106). Furthermore, **BMP2** overexpression appears sufficient to boost cardiac regeneration in zebrafish (106). Nevertheless, the ability of BMP2 in inducing cycle re-entry of mammalian neonatal cardiomyocytes (rat model) is currently controversial (106, 107). BMP2 administration in adult infarcted mice reduces cardiomyocyte apoptosis and scar size, protecting cardiomyocytes from oxidative stress and hypoxia, although potential effects on cardiomyocyte proliferation were not evaluated and deserve further investigations (108, 109).

**BMP10** is essential for maintaining cardiac growth during cardiogenesis in murine models (110). Mechanistically, BMP10 promotes the production of the transcription factor **Tbx20** by inducing its promoter activity through a Smad binding site (111). In turn, cardiomyocyte-specific Tbx20 gain of function, beginning in fetal development, maintains cardiomyocytes in an immature proliferative status, characterized by fetal gene expression and smaller, cycling, mononucleated cells, by inducing BMP2/pSmad1/5/8 and to a lesser extent PI3K/AKT/GSK3β/β-catenin (107). Importantly, intramyocardial injection of BMP10 increases cell cycle activity of adult cardiomyocytes, and its delivery by a sponge scaffold for 12-weeks in an infarcted rat model enhances cardiomyocyte progression to the S-phase, cell re-entry and cytokinesis, and improves cardiac function (112). Other BMP ligands, such as BMP14 and BMP7, were suggested as positive regulators of cardiac repair, even though the documented effects are independent of cardiomyocyte proliferation. *In vivo* ablation of BMP14 (also known as GDF5) results in increased cardiomyocyte apoptosis, fibrosis and adverse cardiac remodeling after myocardial infarction in adult mice (113). BMP7 exerts anti-inflammatory and anti-fibrotic properties (114, 115) [reviewed by Aluganti Narasimhulu and Singla (77)]. Conversely, other BMPs appear to exert an opposite role. It is the case of **BMP4**, which induces hypertrophy and apoptosis in cultured cardiomyocytes (116).

TGFβ1, TGFβ2, and TGFβ3, pleiotropic factors belonging to the transforming growth factor beta (**TGFβ**) superfamily, have emerged as crucial mediators of multiple cellular responses in the infarcted myocardium, including cardiac reparative, inflammatory, angiogenic, and fibrotic responses [reviewed by Frangogiannis (117), Hanna and Frangogiannis (118) and Sorensen and colleagues (119)]. The inhibition of TGFβ receptor 1 (**TGFBR1**) activity by SB-431542 was reported to reduce the number of proliferating cardiomyocytes in zebrafish embryos (120). Intriguingly, robust activation of TGFβ/SMAD3 signaling has been documented during zebrafish heart regeneration, as evidenced by upregulation of TGF ligands (tgfb1a, tgfb1b, tgfb2, and tgfb3), receptors (alk5a known as Tgfbr1, and alk5b known as Tgfbr1b) and the downstream effector SMAD3 (10, 121). Furthermore, inhibition of TGFβ/SMAD3 signaling reduces cardiomyocyte cell cycle activity and abolishes heart regeneration in adult zebrafish upon cardiac injury (10, 121). Interestingly, opposite results have been obtained in mammals. Indeed, the administration of TGFβ has been documented to inhibit the proliferation of neonatal rat cardiomyocytes and suppress the mitogenic effect of growth factors such as bFGF or IGFs (122). Transgenic mice overexpressing TGFβ1 display increased cardiomyocyte size and cardiac hypertrophy accompanied by interstitial fibrosis (123). Moreover, administration of TGFβ inhibitor (SB-431542) robustly induces the proliferation of human iPS-derived cardiomyocytes, if combined with a Wee1 inhibitor, and overexpression of CDK4 and cyclin D1 (47). Another member of the TGFβ superfamily, known as **Myostatin**, was found able to inhibit proliferation of dividing fetal and neonatal rat cardiomyocyte by blocking the G1-S phase transition (124). The opposite role of TGFβ signaling in the modulation of cardiomyocyte replication of lower vertebrates versus mammals deserves further investigation.

**Sonic hedgehog (Shh)** is a ligand of the hedgehog family, which has been mainly implicated in the formation of coronary vasculature and reported to modulate cardiac regeneration and repair [reviewed by Wang and colleagues (125)]. In zebrafish embryos, administration of Shh agonist (SAG) or antagonists (CyA), respectively increases or decreases the number of proliferating cardiomyocytes (120). Moreover, hedgehog signaling is required for myocardial regeneration in zebrafish, further increasing cardiomyocyte proliferation (120). Shh ligand expression and activation of downstream pathways are observed during heart regeneration after cardiac injury in neonatal mice, but not 1 week after birth, when mice are no longer able to regenerate their hearts (126). Finally, genetic or pharmacological augmentation of Shh signaling within the first week of postnatal life in the mouse model has been shown to improve heart regeneration, whereas its inhibition impairs the regenerative response (126). Even if cardiomyocyte proliferation has not been analyzed, gene therapy with Shh after acute and chronic myocardial ischemia in adult mammals results in enhanced neovascularization, reduced fibrosis, and augmented cardiac function (127).

Several **pro-inflammatory cytokines**, such as Interleukin-1β (IL1β), Interleukin-33 (IL33), Interleukin-6 (IL6), Oncostatin (OSM) and TNF-related weak inducer of apoptosis (TWEAK), can induce cardiomyocyte dedifferentiation and/or proliferation, in most cases promoting a beneficial effect in the short run. Indeed, cardiomyocyte dedifferentiation physiologically protects the heart after acute damage. However, in the long run, pro-inflammatory cytokines lead to chronic inflammation, fibrotic disorders, adverse remodeling and/or heart failure. For example, the administration of **IL1β**, which is upregulated upon cardiac injury in neonatal mice (128), induces neonatal cardiomyocyte proliferation (89, 129). However, IL1β is also responsible for profibrotic signaling and cardiomyocyte apoptosis. Its blockage, through platelet microparticles armed with selective antibodies, prevents adverse cardiac remodeling inhibiting cardiomyocyte apoptosis (130). In contrast, **IL33**, another member of the IL-1 superfamily, is among the pro-regenerative factors produced by Treg cells (described in the “*Maternal factors*” section).

In addition, pro-inflammatory cytokines of the IL6 family, including **IL6** and **Oncostatin M (OSM)**, were found elevated in the acute response to cardiac injury. OSM triggers dedifferentiation of cardiomyocytes, as demonstrated by the reduction of sarcomere structure and reactivation of fetal sarcomere components (such as alpha-SMA) and stem cell markers (such as Runx1 and Dab2) (131), physiologically protecting the heart after acute damage. However, in the long run, OSM-induced cardiomyocyte dedifferentiation leads to adverse remodeling and heart failure (131) [reviewed by Fontes and colleagues (132)]. Intriguingly, OSM administration, acting through its receptor (OSMR) and the co-receptor **gp130 (glycoprotein 130)**, also induces cardiomyocyte proliferation in neonatal mice, and synergizes with other mitogenic stimuli such as fibroblast growth factor 2 (FGF2) or adenovirus-induced E2F2 (131). Conditional overexpression of gp130, triggers cardiomyocyte replication and heart regeneration in juvenile and adult mice, via Src-mediated YAP activation (73). IL6 knock out neonatal mice fail to regenerate the heart, whereas IL6 overexpression results in enhanced proliferation of neonatal cardiomyocytes (128, 133). Mechanistically, the pro-regenerative effect of IL6 appears mediated by STAT3 signaling, which also is required for neonatal heart regeneration in mice (128, 133). However, no study thus far evaluated the potential ability of IL6 in inducing cardiomyocyte replication and heart regeneration in the adult stage.

Inflammatory cytokines of the tumor necrosis factor (TNF) ligand family, including Tnfsf11 (also known as **RANKL**), a cytokine secreted by Tregs already described in the “*Maternal factors*” section and Tnfsf12 (also known as **TWEAK**), were also found to positively regulate cardiomyocyte proliferative ability. TWEAK has been enlightened as a positive regulator of neonatal rat cardiomyocyte mitosis through fibroblast growth factor-inducible molecule 14 **(**FN14) receptor. However, early postnatal downregulation of **FN14** restrains TWEAK mitogenic potential in adult cardiomyocytes (134). Nevertheless, adenoviral expression of FN14 enables efficient induction of cell cycle re-entry in adult cardiomyocytes after TWEAK stimulation (134).

A few **anti-inflammatory cytokines**, such as Interleukin-4 (**IL4**, which is secreted by M2 macrophages described in the section entitled “*Cardiac cell populations*”) and Interleukin-13 (**IL13**, a cytokine with anti-inflammatory activities but mediator of allergic inflammation), were suggested to exert a positive effect on cardiomyocyte proliferation. Interleukin-13 (IL13) stimulates neonatal cardiomyocyte replication *in vitro* by activation of IL13Ra1, and to a lesser extent IL4Ra, and downstream pathways, such as STAT6 and STAT3/Periostin (135), ERK and AKT (136). Furthermore, IL13 knock out mice display reduced cardiomyocyte cell cycle activity and impaired cardiac regeneration upon cardiac apex resection at the neonatal stage (136). However, the potential ability of IL13 in inducing cardiomyocyte proliferation and cardiac regeneration in the adult stage remains unexplored.

As described in the “*Maternal factors*” section, other cytokines secreted by Tregs, including Cystatin F (**Cst7**), and Fibrinogen-like 2 (**Fgl2**), were demonstrated to trigger cardiomyocyte proliferation and heart regeneration (50).

Administration of **Follistatin-like 1** (**FSTL1**), an epicardial-secreted cardiac mitogen, through an epicardial patch, improves survival, and sustains cardiac function in infarcted mouse and swine models, by promoting cell cycle re-entry and division of pre-existing cardiomyocytes (137). Some angiogenic factors were also demonstrated to induce myocyte proliferation. For example, cardiac overexpression of **VEGF (vascular endothelial growth factor)** paralog Vegfaa induces cardiac muscle hyperplasia in adult zebrafish, although inhibiting regeneration after injury, suggesting that spatio-temporal control of this factor is required (138). Overexpression of VEGF and **angiopoietin-1 (Ang1)** by adeno-associated viral vectors, in addition to improve angiogenesis, promotes cardiomyocyte cell cycle re-entry in infarcted swine models (139).

Finally, mutation of the **Ciliary Neurotrophic Factor (CNTF)** has been reported to impair cardiomyocyte proliferative response in injured zebrafish hearts, whereas CNTF injection facilitates cardiac regeneration (140).

### Extracellular Matrix

The cardiac extracellular matrix (ECM) is a highly dynamic network of fibers comprised of matrix proteins in which cardiac cells, including cardiomyocytes, reside. It is continuously remodeled in response to environmental stimuli, aging, and pathological conditions, to support a wide variety of cellular responses. During the early postnatal development, the cardiac matrix changes its mechanical properties from a high hydrated structure, enriched with fibronectin, hyaluronic acid, and proteoglycans, to a stiffer structural network, enriched with collagen I, and laminin, thus supporting the strength of the cardiac muscle (141). Importantly, the increased rigidity of the heart after birth mechanically influences cardiomyocyte morphology and behavior, contributing to their cell cycle withdrawal. Indeed, rigid substrates interfere with rat and mouse cardiomyocyte cytokinesis, without affecting karyokinesis (nuclear division), thus leading to binucleation (142). On the other hand, softer substrates trigger cardiomyocyte rounding and cell division, coupled with a partial cardiomyocyte dedifferentiation process as documented by downregulation of sarcomere proteins (142).

A key pathway in matrix-stiffness mechano-transduction is the **Hippo pathway**, a well-known regulator of organ growth (143, 144). Importantly, the Hippo pathway has emerged as a key regulator of heart regeneration. If activated in the cardiac tissue, it drives phosphorylation of **YAP (Yes-associated protein)**, thus preventing its translocation into the nucleus, in turn restraining cardiomyocyte proliferation in postnatal life [reviewed by Wang and colleagues (145)]. Inactivation of the Hippo signaling, by deletion of scaffold proteins, such as **Sav1 (Salvador homologue 1 protein)**, or downstream mediators, such as **Lats1** and **Lats2 (large tumor suppressor homologue 1 and 2)**, or alternatively constitutive expression of active YAP, in adult cardiomyocytes, have been shown to stimulate cardiomyocyte proliferation, reduce the scar size, and improve heart function in infarcted mouse models (146–149) [reviewed by Wang and colleagues (145)].

The stiffness is not the only way by which the extracellular matrix may impact on cardiomyocyte replicative and regenerative ability. Indeed, it has been demonstrated in the mouse model that the changes in the composition of the cardiac extracellular matrix during the early postnatal period influence cardiomyocyte growth and differentiation. For example, the proteoglycan **Agrin** is physiologically downregulated during the early postnatal cardiac development, contributing to the loss of cardiomyocyte proliferative potential (150). Agrin interacts with the dystrophin-glycoprotein complex (DGC), connecting the ECM to the F-actin cytoskeleton, through **Dag1 (dystroglycan 1)**. Administration of Agrin following myocardial infarction in juvenile and adult mice is sufficient to destabilize the cardiomyocyte cytoskeleton and facilitate cell cycle re-entry and cell division in the peri-infarcted region by activating downstream mediators such as the **extracellular signal-regulated kinase (ERK)** signaling and destabilizing YAP-Dag1 interaction, leading to **YAP** release and translocation into the nucleus (150). A single local delivery of recombinant human Agrin has been documented to enhance cardiomyocyte proliferation, improve cardiac function and reduce adverse remodeling, fibrosis, and infarct size in preclinical swine models (151).

Some members of the **fibronectin 1** family (fn1 and fn1b), the main components of the extracellular matrix, are produced and deposited after cardiac damage in the zebrafish model and are essential for the regenerative process (152). Fibronectin 1 (Fn1) and **collagen III**, produced by embryonic fibroblasts (described in the “*Cardiac cell populations*” section), have also been shown to induce mammalian cardiomyocyte proliferation (80). **β1-integrin** appears to be required for the proliferative response induced by embryonic fibroblast-secreted factors, and ventricular cardiomyocyte-specific deletion of β1-integrin in mice reduces myocardial proliferation and impairs ventricular compaction (80).

**Periostin**, a secreted extracellular matrix protein, promotes adult rat cardiomyocyte proliferation via activation of **αV**, **β1**, **β3**, and **β5 integrins** and downstream activation of **PI3K/AKT** (but not ERK) pathway (153). After myocardial infarction in adult rats, Periostin induces cardiomyocyte cell cycle re-entry and mitosis, improves ventricular remodeling and reduces infarct size (153). However, Periostin is also responsible for the recruitment of activated fibroblasts in the mouse model (154) and promotes extensive cardiac fibrosis in remote regions in infarcted swine models (155), thus its administration as a strategy for inducing cardiac regeneration is dampened.

**Connective tissue growth factor (CTGF)**, also known as communication network factor 2a (Ccn2a), is a matricellular protein that is synthesized and secreted from endocardial cells after cardiac injuries (156). In zebrafish, CTGF has been reported necessary for heart regeneration by inducing cardiomyocyte proliferation and infiltration (156). CTGF triggers cardiomyocyte cell cycle activity also in neonatal mammals (135), however, its potential impact on adult cardiomyocyte proliferation and heart regeneration has not been explored thus far.

Finally, the extracellular matrix protein **Matrilin2 (Matn2)** is among the factors secreted by Tregs that trigger cardiac proliferation and heart regeneration (50) (described in the “*Maternal factors*” section). Thus, administration of extracellular matrix components, or modulation of the downstream signaling pathways, might be a promising approach for heart regeneration.

### Cell-to-Cell Contact

**Notch ligands** are transmembrane proteins, therefore the signaling is activated when the cell expressing the ligand is adjacent to the cell expressing the notch receptor. Ligand binding leads to cleavage and release of the Notch intracellular domain (NICD), which then travels to the nucleus to regulate transcriptional complexes. The Notch signaling plays an essential role for trabeculation of the ventricular myocardium during mammalian cardiac development, as well as in heart health (157) [reviewed by MacGrogan and colleagues (158)]. The inhibition of Notch signaling has been shown to suppress the proliferation and to induce apoptosis of mammalian immature neonatal cardiomyocytes, highly expressing the notch receptor **Notch1**. However, Notch 1 expression levels decline during cardiac maturation (159). Enforced activation of the Notch signaling by constitutive expression of the active intracellular domain of Notch1 (N1 ICD), or stimulation with the ligand **Jagged1**, boosts the proliferation of immature cardiomyocytes (159).

### Systemic Hormones

Hormones are signaling molecules that act distant from their site of production. Interestingly, some hormones belonging to steroid, eicosanoid, amino acid-derived, and protein subclasses have been investigated for their ability to modulate cardiomyocyte proliferation and heart regeneration.

**Steroid hormones** can be grouped into types according to the receptors to which they bind, namely glucocorticoids, mineralocorticoids, androgens, estrogens, progestogens and Vitamin D derivatives. Glucocorticoids and mineralocorticoids are typically synthesized in the adrenal cortex (hence they are also known as corticosteroids), whereas androgens, estrogens, and progestogens are sex steroids, typically synthesized in the gonads or placenta. All of them are released into the circulatory system.

**Glucocorticoids (GCs)** exert most of their actions through the **Glucocorticoid Receptor (GR)**, and in some tissues or conditions through Mineralocorticoid Receptor (MR). In zebrafish, stress-induced cortisol secretion blocks cardiomyocyte proliferation and cardiac regeneration after cryoinjury (160). In mammals, circulating active glucocorticoid levels physiologically rise shortly before birth in preparation for postnatal life by promoting the maturation of the lungs and other organs. During late gestation, endogenous glucocorticoids were shown to induce the maturation of fetal cardiomyocytes via activation of GR receptor (161), whereas their impact on fetal cardiomyocyte proliferation is currently controversial (162, 163). A few studies reported the adverse side-effects of synthetic glucocorticoid therapy in preterm infants resulting from impaired cardiomyocyte proliferation and endowment (164–166). Importantly, a role for physiological glucocorticoids in postnatal cardiomyocyte growth and regenerative plasticity has been recently suggested in the mouse model. Indeed, a physiological increase in GR activation by endogenous glucocorticoids in the early postnatal development concurs to restrain the proliferative ability of neonatal cardiomyocytes [pre-publication by Pianca and colleagues (167)]. Cardiomyocyte-specific GR ablation (GR-cKO) appears sufficient to boost neonatal cardiomyocyte proliferation and to delay the early postnatal transition from hyperplastic to hypertrophic growth along with the maturation of myofibrils-mitochondria organization [pre-publication by Pianca and colleagues (167)]. Further analysis unveiled that GR ablation increases cardiomyocyte replication by regulating the energetic metabolism, favoring glucose catabolism over fatty acid oxidation [pre-publication by Pianca and colleagues (167)]. However, in later stages of postnatal life, no differences in cardiomyocyte proliferation rate were reported in GR ablated compared to control mice (168). Nevertheless, upon myocardial infarction, cardiomyocytes in GR ablated juvenile and adult mice are facilitated to re-enter into the cell cycle and divide, leading to regeneration of the lost cardiac tissue along with reduced scar formation [pre-publication by Pianca and colleagues (167)]. Altogether these results support a model where increased activation of GCs/GR axis restrains the regenerative plasticity of cardiomyocytes.

The lower incidence of cardiovascular disease and mortality rate in women compared to men of similar age, along with the increased occurrence in women after menopause, have suggested that gender-related differences in **sex steroid hormones** (in particular estradiol) play a key role in the development and evolution of cardiovascular disease [reviewed by Vitale and colleagues (169)]. Studies on lower vertebrates have demonstrated that sexual dimorphism reflects also a dimorphic cardiac regenerative response. Indeed, female zebrafish display higher rates of cycling cardiomyocytes in both cryoinjured and uninjured regenerating hearts compared to males (170). Furthermore, exposure to **estrogen** accelerates male zebrafish regeneration after damage, by enhancing cardiomyocyte dedifferentiation and proliferation. Instead, exposure to tamoxifen, an estrogen receptor antagonist, delays female heart regeneration (170). Nevertheless, the role of estrogens in cardiac regenerative plasticity in mammals remains so far unknown. Recently, **progesterone** has emerged as a mediator of sex-dependent transcriptional programs during cardiomyocyte maturation (171). Interestingly, progesterone supplementation has been suggested to increase cardiomyocyte proliferation and heart regeneration after myocardial infarction in a progesterone receptor-dependent manner, by increasing YAP expression and signaling (172).

**Vitamin D** has been reported to regulate cardiomyocyte proliferation both in zebrafish and mouse models. In zebrafish, Vitamin D promotes cardiomyocyte cycling and tissue regeneration, and this process requires intact Erbb2 signaling (173). In contrast, the administration of Vitamin D to cultured mouse cardiomyocytes has been reported to induce both anti-proliferative (168, 174–176) and pro-proliferative effects (173). Furthermore, the deletion of the Vitamin D receptor appears not sufficient to prolong the postnatal cardiomyocyte proliferative window in the mouse model (168). A potential explanation of these conflicting results could be that the effects of Vitamin D on cell proliferation may be context-dependent and/or concentration-dependent. Despite these discrepancies, Vitamin D supplementation was proved to reduce ventricular remodeling and improve cardiac function in heart failure patients [metanalysis of several clinical trials by Zhao and colleagues (177)].

Among eicosanoid hormones, **Prostaglandin E2 (PGE2)**, a principal mediator of inflammation, is upregulated in the injured zebrafish heart and the suppression of its production by administration of Cox2 inhibitors reduces cardiomyocyte proliferation in response to cardiac injuries (178).

Among amino acid-derived hormones, **thyroid hormones**, namely **triiodothyronine (T3)** and **thyroxine (T4)**, gained attention in the context of cardiomyocyte proliferative ability. The analysis of 41 different species unveiled an inverse correlation between cardiomyocyte diploid content (index of mitogenic potential) and plasma T4 levels. Interestingly, T4 levels raise soon after birth, coincident with cardiomyocyte withdrawal from the cell cycle and binucleation/polyploidization. Moreover, inactivation or cardiomyocyte-specific ablation of thyroid hormone receptor-α (TRα) counteracts mammalian cardiomyocyte polyploidization, increasing the number of diploid proliferating cells and therefore the regenerative potential (179). Furthermore, T3 administration to fetal cardiomyocytes promotes their maturation while suppressing their proliferation (180, 181) and reduces cardiomyocyte replication at the neonatal stage (122). In contrast, a surge in T3 levels has also been reported to initiate a brief but intense proliferative burst of predominantly binuclear cardiomyocytes during pre-adolescence (182), although the existence of this burst was disproved (183, 184).

**Melatonin**, an amino acid-derived hormone produced by the pineal gland, exerting a protective role against oxidative stress, apoptosis, and inflammation after cardiac injury, has also been documented to induce cardiomyocyte proliferation after myocardial infarction in the mouse model (185). The suggested mechanism involves the activation of the melatonin receptor and regulation of the miR-143-YAP axis (185) (further described in “*miRNAs*” section).

Among protein hormones, insulin-like growth factor signaling has been demonstrated to play a role in cardiomyocyte regenerative ability. During embryonic heart development, **Insulin-like growth factor 2 (IGF2)** appears to be the most prominent mitogen made by epicardial cells (186). The expression of the zebrafish homolog Igf2b was found upregulated during zebrafish heart regeneration, and inhibition of its receptor IGF1R blocks cardiomyocyte proliferation during heart development and regeneration (187). Administration of IGF signaling agonist (NBI-31772) or antagonist (NVP) respectively boosts or reduces cardiomyocyte proliferation in zebrafish embryos (120). Furthermore, IGF signaling is required for cardiomyocyte replication during myocardial regeneration in zebrafish (120). As described in the “*Maternal factors*” section, IGF2 is among the factors secreted by maternal Treg cells during gestation, inducing cardiomyocyte proliferation and heart regeneration in adult mice (50).

The administration of low-dose IGF1 induces beneficial effects on remodeling in post-infarct patients, despite not improving heart function (188). Intramyocardial delivery of **Insulin-like growth factor 1 (IGF1)** together with **Hepatocyte growth factor (HGF)**, through hydrogel or saline injection, enables endogenous cardiac repair on infarcted swine hearts, leading to the generation of new immature cardiomyocytes (189). In this regard, intracoronary administration of adenovirus carrying the HGF gene modestly reduces heart dilation and improves heart function in heart failure patients (190).

### Signaling Cascades

A large number of growth factors and cytokines transduce their effects via the RAS-mitogen activated protein (MAP) kinase signaling (also known as **Ras-Raf-MEK-ERK pathway**). The key role of ERK signaling in triggering cardiomyocyte dedifferentiation and proliferation has emerged in multiple studies, for example, downstream to NRG1/ERBB2 axis (102), Agrin (150), OSM (131), IL13 (136), and IGF signaling (186). Intriguingly, the suppression of **Dual specificity phosphatase 6 (DUSP6)**, which antagonizes the activation of the MAPK cascade, results in increased myocyte proliferation during embryonic and early postnatal development, as well as enhanced cardiac regeneration in zebrafish (191) and mice (192).

Proinflammatory cytokines (such as IL-1 and TNF-α), some mitogens, cellular stress (including UV irradiation, heat shock, and high osmotic stress), lipopolysaccharide, and protein synthesis inhibitors, may activate **P38 mitogen-activated protein (MAP) kinase signaling**, which has been enlightened as a negative regulator of cardiomyocyte division. P38 inversely correlates with cardiac growth during mammalian embryonic development (89). Its *in vivo* activation inhibits fetal cardiomyocyte DNA synthesis, whereas cardiac-specific ablation of p38α enables neonatal cardiomyocyte proliferation (89). Furthermore, pharmacological inhibition of p38 is sufficient to stimulate replication of adult ventricular cardiomyocytes (from 12-weeks-old rats), upregulating genes involved in cell cycle progression, mitosis and cytokinesis (including cyclin A2, cyclin B and aurora B) (89). P38 inhibition also boosts the mitogenic effect of growth factors, such as FGF1, NRG1 and IL1β (89, 90). After myocardial infarction in adult mice, combinatorial therapy with p38 inhibitor and FGF1 has been shown to induce cardiomyocyte proliferation and cardiac tissue regeneration, reduce scar formation and improve cardiac function (90). Interestingly, p38 MAP kinase inhibition alone is not able to boost heart function despite increased cardiomyocyte mitosis (90). A clinical trial to assess the safety and efficacy of losmapimod, a p38 inhibitor, has been initiated, however, it was stopped when non-encouraging trials of the Tumor Necrosis Factor-α (TNF-α)-targeting [whose cardio-depressant action is induced by activation of p38 (193)] in heart failure patients were reported (194) [reviewed by Javed and Murtaza (195)].

In the mouse model, ablation of **cardiac troponin I-interacting protein kinase (TNNI3K)**, a cardiomyocyte-specific MAPKKK, results in an increase of mononuclear diploid cardiomyocytes, facilitating heart regeneration after injury (196, 197). On the other hand, TNNI3K overexpression in zebrafish induces cardiomyocyte polyploidization and impairs heart regeneration (196).

Several cytokines activate the **Jak-STAT signaling**, which plays an important role in the maintenance of cardiac homeostasis and takes part in the acute inflammation occurring after heart injuries [reviewed by Barry and colleagues (198)]. In zebrafish, Jak1/STAT3 pathway is activated after cardiac injury (199). Furthermore, cardiomyocyte-specific deletion of **STAT3**, a downstream effector of inflammatory cytokines, such as IL6 and OSM, reduces cardiomyocyte proliferation during the injury-induced cardiac regenerative response in zebrafish (199) and neonatal mice (128). Thus, STAT3 is essential for heart regeneration. In addition, therapeutic activation of STAT3 by IL11 administration was shown to reduce fibrosis and attenuate cardiac dysfunction after myocardial infarction (200).

The administration of a **Glycogen synthase kinase 3 beta (GSK3β)** inhibitor, which leads to **β-catenin** nuclear accumulation, stimulates neonatal and adult cardiomyocyte dedifferentiation and proliferation (201). Moreover, germ-line deletion of GSK3β results in hyperproliferation of cardiomyocytes (202). However, in the latter model, no difference in β-catenin localization could be observed, suggesting that GSK3β may modulate cardiomyocyte replication in a β-catenin independent manner. Furthermore, inducible cardiomyocyte-specific deletion of GSK3-β stimulates cardiomyocyte mitogenesis and exhibits a protective role against cardiac remodeling after myocardial infarction (203). The administration of **N-cadherin** antibodies, which induce the release of sequestered β-catenin from adherent junctions, promotes cardiomyocyte cell cycle re-entry (204). Similarly, adenoviral induced overexpression of β-catenin in the cardiac tissue results in increased cardiomyocyte cell cycle activity and reduced myocardial infarct size, even if cardiomyocyte binucleation and hypertrophy, without an evident increase in cardiomyocyte number, have been documented (205). Intriguingly, the accumulation of β-catenin has been observed upon constitutive activation of ERBB2 signaling, specifically mediating cardiomyocyte dedifferentiation (102). Ablation of **lipoprotein-related receptor protein LRP6** (a coreceptor interacting with Frizzled receptor in Wnt/β-catenin signaling) in infarcted mouse hearts stimulates robust regenerative processes through the proliferation of pre-existing cardiomyocytes via a β-catenin independent mechanism, involving **ING5 (inhibitor of growth family member 5)**/p21 (206).

Finally, knockdown of the E3 ubiquitin ligases **Cbl** and **Itch** induces neonatal rat cardiomyocyte proliferation *in vitro* (207).

### Transcription Factors

The decline of the proliferative and regenerative ability of cardiomyocytes in early postnatal development has been reported to be regulated by several transcription factors.

**GATA4 (GATA binding protein 4)** expression increases in cycling cardiomyocytes during heart regeneration in zebrafish (13). In neonatal mice ablation of GATA4 impairs cardiomyocyte proliferation and cardiac regeneration (92, 208). Furthermore, GATA abundance in the murine cardiac tissue decreases in the early postnatal period, and its overexpression by adenoviral gene transfer improves cardiac regeneration in 7-day-old mice (208). A suggested mechanism by which GATA4 exerts this regenerative effect is the increased expression of regenerative growth factors and cytokines, such as IL13 or FGF16, although the latter one is controversial (92, 208).

**Meis1 (myeloid ecotropic viral integration site 1)**, whose abundance in the cardiac tissue modestly raises in the early postnatal period, is a crucial mediator of cardiomyocyte cell cycle arrest (209). Indeed, cardiomyocyte-specific deletion of Meis1 extends their proliferation, whereas its overexpression limits neonatal heart regeneration following myocardial infarction by upregulating cyclin-dependent kinase (CDK) inhibitors p15, p16 and p21 (209). Double knockout of Meis1 and **Hoxb13 (Homeobox B13)**, a cofactor of Meis1, reactivates cell cycle activity in adult cardiomyocytes, induces sarcomere disassembly and improves cardiac function following myocardial infarction (210).

Recently, combinatorial knockdown of **Meis2** (a member of the same family) and **Retinoblastoma (Rb1)**, through hydrogel-based delivery of small interfering RNAs in adult rats, was reported to significantly increase cardiomyocyte proliferation, to reduce infarct size and to improve cardiac function post-myocardial infarction (211).

**Pitx2 (Paired-like homeodomain 2)** has been reported to exert a key role in myocardial regeneration of neonatal and adult mice. Indeed, it is required for neonatal cardiac regeneration and sufficient to trigger adult myocardial regeneration in the mouse model (212). Mechanistically, it has been shown that Pitx2 promotes the expression of ROS scavengers, protecting cells from oxidative damage (212). Interestingly, Pitx2 is induced during heart regeneration triggered by Hippo deficiency (212) and its expression is stimulated by the transcription factor **Nrf2 (nuclear factor erythroid 2–related factor 2)**, whose ablation also impairs neonatal cardiac regeneration (212).

Multiple studies over time have pointed out the role in mammalian cardiac regeneration of **E2F family** members, transcription factors known to regulate cell cycle progression. Adenoviral delivery of **E2F1** triggers S-phase entry of adult rat cardiomyocytes *in vitro*, however, stimulating cell death (213, 214). Interestingly, **p53** ablation boosts E2F1-induced cardiomyocyte proliferation, despite not preventing apoptosis (214). Overexpression of **E2F2, E2F3**, and **E2F4** is sufficient to enhance the proliferation of neonatal cardiomyocytes *in vitro* (215) and, in the case of E2F2, also in terminally differentiated cardiomyocytes *in vivo* (216). Similar to E2F1, E2F3 overexpression was also associated with cell death (215). Instead, contrasting data have been obtained for the apoptotic response induced by **E2F2** and **E2F4**, with initial studies demonstrating a reduction in cell death upon E2F2 and E2F4 overexpression in cultured neonatal cardiomyocytes (215), and more recent studies describing an increase in apoptosis of cultured adult mammalian cardiomyocytes (217). Interestingly, co-expression of E2F2 and BEX1 [Brain Expressed X-Linked (Bex)] was demonstrated as a strategy to induce cardiomyocyte cell cycle activity, without cell death (217).

The transcriptional repressor **REST (transcriptional repressor element-1 silencing transcription factor)** can also trigger the proliferation of cultured cardiomyocytes (218). REST is required for normal embryonic cardiac development and neonatal regeneration upon injury, sustaining cardiomyocyte cell cycle activity by repressing the cell cycle inhibitor gene p21 (218).

Recently **Klf1 (Krüppel-like factor 1)** has been reported to be required for heart regeneration in zebrafish stimulating epigenetic and metabolic remodeling (219).

Other transcription factors that emerged as regulators of cardiomyocyte regenerative potential, such as **YAP**, **GR, HIF1α**, **SMADs**, **Tbx20**, **p53**, and **Jarid2** were described in other sections of this review.

### Epigenetic Regulations

The remodeling of the epigenetic landscape has also been linked to cardiomyocyte regenerative ability. RNA sequencing analysis of cardiomyocytes unveiled a differential transcriptomic framework at various stages of postnatal life in healthy and infarcted mammals (220). The epigenetic modulation of specific genes, in particular those involved in chromatin compaction and cell cycle, has been suggested to contribute to the proliferative inability of terminally differentiated cardiomyocytes (220). Furthermore, chromatin-remodeling proteins contributing to mantain a fetal-like status are switched off in adult cardiomyocytes. It is the case of **Brg1 (Brahma-related gene-1)**, which promotes proliferation of embryonic mammalian cardiomyocytes by maintaining Bmp10 expression and repressing p57 (221). In addition, by interaction with HDAC (histone deacetylase) and PARP (poly ADP-ribose polymerase), Brg1 represses Myh6 (α-myosin heavy chain, mainly expressed in adult cardiomyocytes) and activates Myh7 (β-myosin heavy chain, mainly expressed in embryonic cardiomyocytes), thus controlling myosin heavy-chain switching during embryonic/neonatal development, as well in adulthood under cardiac stress-induced hypertrophy (221). Interestingly, transgenic inhibition of Brg1 in the zebrafish model impairs cardiomyocyte proliferation and myocardial regeneration by repressing CDK inhibitors, such as cdkn1a and cdkn1c (222).

Recent studies have unveiled a role in cardiomyocyte regeneration for **ALKBH5 (α-ketoglutarate-dependent dioxygenase alkB homolog 5)**, a N^6^-methyladenosine eraser of messenger RNAs. Indeed, ALKBH5 expression levels decline in postnatal development and its overexpression promotes cardiomyocyte replication and cardiac regeneration following myocardial infarction in juvenile and adult mice, by increasing YAP translation (223). In contrast, knock out of **methyltransferase-like 3 (METTL3)**, a N^6^-methyladenosine writer, whose expression levels raise postnatally, induces cardiomyocyte cell cycle re-entry, reduces scar size and boosts cardiac function after myocardial infarction, by regulating the miR-143-YAP axis (224).

Finally, ablation of the DNA methylase **Dnmt3a**, which is significantly downregulated after injury-induced cardiac regeneration, triggers neonatal rat cardiomyocyte proliferation *in vitro* (207).

### miRNAs

Several miRNAs were found to regulate cardiomyocyte proliferative and regenerative ability. High-throughput screening in neonatal rat cardiomyocytes led to the identification of 40 miRNAs able to stimulate karyokinesis and cytokinesis in neonatal cardiomyocytes (225). *In vitro* administration of two of them, **miR-590** and **miR-199a**, is sufficient to enhance the proliferation of adult rat cardiomyocytes (225). Importantly, cardiomyocyte proliferation was also observed after their delivery *in vivo* by intracardiac injection of lipid transfection complexes or by adenoviral vectors, leading to cardiac regeneration after myocardial infarction in the mouse model (225). The pro-regenerative efficacy of miR-199a has been also validated in larger animal models upon cardiac injury (infarcted swine), however, arrhythmic death after the persistent expression was reported (226).

**miR-1825** has been reported to induce a pro-mitotic effect on adult cultured rat cardiomyocytes, along with alterations in the electron transport chain, and a decrease in mitochondrial numbers, oxygen species and DNA damage (227). Importantly, intra-cardiac delivery of miR-1825 enhances the proliferation of adult cardiomyocytes in the peri-infarcted region. Multiple pathways seem to mediate the pro-regenerative effects of miR-1825, including the upregulation of miR-199a, which in turn repress its targets p16, Rb1, and Meis2 (227).

A few miRNAs, including **miR-548c**, **miR-509**, and **miR-23b**, have been documented to stimulate the proliferation of adult cardiomyocytes through inhibition of Meis1 (228).

Overexpression of members of miR-17-92 cluster, such as **miR-17-92** (by employing α-MHC-Cre transgenic mice) (229) and **miR-19a/19b** (delivered through adeno-associated viruses) (230), is sufficient to enhance the proliferation of embryonic, postnatal, and adult cardiomyocytes (229). Intra-cardiac injection of miR-19a/19b promotes a robust regeneration process of the infarcted cardiac tissue and boosts heart function (230). Among the suggested mechanisms for the pro-proliferative/regenerative effects of miR-17-92 members, it has been suggested the targeting of the oncosuppressor **PTEN** and the repression of the immune and inflammatory injury-induced response (229).

**miR302-367** has been shown to regulate the cell cycle of adult cardiomyocytes by repressing Mst and Lats kinases of Hippo signaling, as well as by altering differentiation and by down-regulating genes involved in fatty acid metabolism (231). Transient expression of miR302-367 promotes mouse cardiac regeneration, avoiding the adverse dedifferentiation and reduced function observed in long-term induction (231).

Interestingly, the pro-proliferative effects induced by several miRNAs, including miR-590, miR-302d, miR-302c, miR-373, miR-1825, miR-1248, miR-18a, miR-33b, miR-30e and miR-199a, were suggested to rely on YAP nuclear translocation, as well as actin polymerization by downregulation of **Cofilin2** (67).

Transgenic mice with cardiac overexpression of **miRNA-204** exhibit cardiomyocyte replication in the embryonic and adult stages. Mechanistically, miRNA-204 induces the degradation of **Jarid2** (jumonji), in turn promoting the expression of several cyclins. Overexpression of Jarid2 impairs cardiomyocyte pro-mitotic effect of miRNA-204 overexpression (232) and reduces embryonic cardiomyocyte proliferation by repressing cyclin D1 expression (233, 234).

Physical exercise has been demonstrated to induce cardiogenesis. Indeed, 2-months-old mice undergoing voluntary wheel running exhibited a greater ability to generate new cardiomyocytes at a projected annual rate of 7.5% compared to 1.63% in sedentary conditions (235). Interestingly, a higher frequency of diploid/mononucleated and proliferating cardiomyocytes was observed in the border zone of post-infarcted mice in exercise (235). The exercise-induced cardiogenesis has been suggested to be mediated by **miR-222**, which increases in response to exercise, and whose inhibition suppresses the mitogenic response (235).

Other miRNAs, including **miR-31a-5p** and **miR-708**, have been found crucial for neonatal cardiomyocyte replication *in vitro* (236, 237). Furthermore, *in vivo* delivery of **miR-708** in lipid nanoparticles was documented to confer cardiomyocyte protection against stress-induced apoptosis, although its role on cardiac regeneration remains unexplored (236).

Some miRNAs may negatively affect cardiomyocyte replication, thus their inhibition may be used to boost cardiac regeneration ability. For example, it has been demonstrated that **miR-99/100** and **let-7** are downregulated upon cardiac injury to facilitate cardiac regeneration in zebrafish (238). In contrast, their expression remains stable in mammals following cardiac damage. Anti-miR-99/100 and anti-let-7 *in vivo* delivery in infarcted mice results in cardiomyocyte dedifferentiation and proliferation, leading to reduced scar size and improved cardiac function (238).

**miR-195**, a member of the miR-15 family, has been identified as a positive mediator of cardiomyocyte cell cycle arrest by blocking the progress through the G2 checkpoint (239). Inhibition of miR-195, via administration of locked nucleic acid (LNA)-modified anti-miRs, from the early postnatal period until adulthood, extends the proliferative window of endogenous cardiomyocytes, regenerating the infarcted heart (239, 240).

Delivery of **miR-34a**, whose expression rises to adult levels within the first postnatal week, suppresses cardiomyocyte proliferation and regeneration in neonatal infarcted hearts (241). Conversely, anti-miR-34a treatment improves cardiomyocyte cell cycle activity and cardiac remodeling post-injury in adult mice (241).

Cardiac-specific overexpression of **miR-128** impairs neonatal and adult cardiomyocyte replication, whereas its ablation allows post-mitotic cardiomyocytes to re-enter the cell cycle driving epigenetic remodeling of pro-mitotic genes, including the chromatin modifier SUZ12, which ultimately triggers repression of CKI p27 (242).

Recently melatonin administration or METTL3 ablation have been shown to downregulate **miR-143**, in turn enhancing the expression of YAP, thus leading to neonatal cardiomyocyte proliferation (185) and heart regeneration (224).

Finally, anti-**miR-29a**, anti-**miR-30a**, and anti-**miR-141** increase neonatal cardiomyocyte cell cycle activity (243).

## Concluding Remarks and Future Directions

In recent years, multiple approaches were suggested to reactivate the cell cycle machinery and regeneration of endogenous cardiomyocytes in the perspective of repairing the damaged myocardium and boosting cardiac function after severe cardiac injuries. In some cases, pre-clinical studies in larger animal models (swine) and clinical trials on post-infarct and/or heart failure patients were also performed, and promising results were documented. In this regard, combinatorial strategies deserve further investigation since they may be more effective. However, several of the suggested approaches still exhibit some limitations and require some precautions. To begin with, some treatments may have a differential response and/or side effects among mammals (for example miR-199a induced arrhythmia in large animals, but not in mice). Furthermore, the tissue specificity of the therapy is quite important to avoid potential side effects. More selective delivery systems are being developed, such as those based on biomaterials (cardiac patches, sponges, hydrogels, etc.), although further improvement in tolerance to immunogenic host responses is required [reviewed by Bar and Cohen (244) and Mei and colleagues (245)].

The delivery system may also have safety issues that should be carefully evaluated (for example, long-term effects induced by adenoviral vectors). Another important issue is the functionality and tissue integration of newly generated myocytes, especially in the strategies resulting in consistent dedifferentiation processes that may lead to altered cardiac function, if persisted. To this end, the duration of the stimulus that promotes cardiomyocyte proliferation is likely a key factor, which requires careful calibration. Additionally, strategies that facilitate re-differentiation of newly generated cardiomyocytes have also been suggested, for example by enhancing cell-cell coupling via overexpression of connexin 43 (246). Other safety issues include a careful evaluation of potential cancerogenic effects, especially for strategies employing genes that are known to play a major role in cancer development. Increased specificity to the cardiac tissue and the transient nature of the stimulus may likely reduce or avoid this problem.

Thus, induction of endogenous cardiomyocyte proliferation represents a promising and flourishing research approach to induce cardiac regeneration after major injuries, although further investigations are required to increase its efficacy and safety.

## Author Contributions

CB and GD'U wrote the manuscript, with inputs and intellectual contribution from the other authors. All authors listed approved the manuscript for publication.

## Funding

This project has been supported by Fondazione Carisbo to GD'U (Grant No: 2020.0389), by Fondazione Luisa Fanti Melloni to GD'U, by Fondazione Cariplo to GD'U (Grant No: GR 2017-0800) and by Ministry of Health - Ricerca Corrente - IRCCS MultiMedica.

## Conflict of Interest

The authors declare that the research was conducted in the absence of any commercial or financial relationships that could be construed as a potential conflict of interest.

## Publisher's Note

All claims expressed in this article are solely those of the authors and do not necessarily represent those of their affiliated organizations, or those of the publisher, the editors and the reviewers. Any product that may be evaluated in this article, or claim that may be made by its manufacturer, is not guaranteed or endorsed by the publisher.
